# Viral gene delivery vectors: the next generation medicines for immune-related diseases

**DOI:** 10.1080/21645515.2020.1757989

**Published:** 2020-05-15

**Authors:** Peter De Haan, Ferdy R. Van Diemen, Miguel G. Toscano

**Affiliations:** a Department of R&D, Amarna Therapeutics B.V, Leiden, The Netherlands; b Department of R&D, Amarna Therapeutics S.L, Sevilla, Spain

**Keywords:** Immune system, immune tolerance, self-antigen, vaccine, cancer, autoimmune disease, alphavirus, sv40, adeno-associated virus, lentivirus, viral vector, gene therapy, immunotherapy, tolerization

## Abstract

Viruses have evolved to efficiently express their genes in host cells, which makes them ideally suited as gene delivery vectors for gene and immunotherapies. Replication competent (RC) viral vectors encoding foreign or self-proteins induce strong T-cell responses that can be used for the development of effective cancer treatments. Replication-defective (RD) viral vectors encoding self-proteins are non-immunogenic when introduced in a host naïve for the cognate virus. RD viral vectors can be used to develop gene replacement therapies for genetic disorders and tolerization therapies for autoimmune diseases and allergies. Degenerative/inflammatory diseases are associated with chronic inflammation and immune responses that damage the tissues involved. These diseases therefore strongly resemble autoimmune diseases. This review deals with the use of RC and RD viral vectors for unraveling the pathogenesis of immune-related diseases and their application to the development of the next generation prophylactics and therapeutics for todays’ major diseases.

## The human immune system

Our immune system ensures homeostasis by protecting us from infection, malignant cell growth and by playing an important role in wound and tissue repair. Today’s major human diseases coincide with aberrant immune responses: cancer patients lack effective immune responses to malignant cells, patients with autoimmune diseases have excessive immune responses to cells of a specific tissue or organ and allergies result from having undesired and exaggerated immune responses to harmless antigens. Innate immunity, involving macrophages, dendritic cells and granulocytes, is the first line of active immunity. Cells of the innate immune system are activated by pathogen-associated molecular patterns (PAMPs) released from infected cells or damage-associated molecular patterns (DAMPs) released from malignant or wounded cells. Additionally, DAMPs are released from damaged body cells as the result of ongoing metabolic and mechanical activity. When PAMPs or DAMPs are registered by innate immunity cells, inflammasomes assemble in the cytoplasm and a local inflammatory response is initiated.^1^ Inflammation is a highly orchestrated cascade of protective local and systemic events aimed at confining a pathogen, stopping malignant cell growth, reducing cell damage and promoting wound repair. The inflammation stops and homeostasis is restored when all necroptotic and proinflammatory cellular components including the PAMPs or DAMPs are removed by cells of the innate immune system.

When an infection is too widespread, the malignancy grows too fast or the wound is too big, the innate immunity cells recruit and activate a second line of immunity: the adaptive immune system. The adaptive immune system consists of two different sets of lymphocytes circulating in the blood and lymphatic system. B lymphocytes (B cells) secrete antibodies and are responsible for humoral immunity, whereas T lymphocytes (T cells) are capable of directly destroying damaged, malignant or infected cells and are responsible for cellular immunity. In contrast to the innate immune system that responds to PAMPs and DAMPs, lymphocytes respond to antigenic peptides (epitopes) derived from self or foreign proteins (antigens), bound to major histocompatibility complex (MHC) molecules in the presence of co-inhibitory or co-stimulatory molecules on the cellular membranes of all body cells.^2^ The lymphocyte population consists of clones, in which each clone bears a different receptor that recognizes a specific MHC-bound epitope. During homeostasis, lymphocytes are in tolerance mode. Peripheral immune tolerance is actively maintained by homeostatic interactions between our body cells, innate immunity cells and lymphocytes, wherein immune responses to self and foreign antigens are actively suppressed. Lymphocytes actively migrate to inflamed areas, locally break the immune tolerance in an antigen-specific manner and start to destroy infected, malignant or damaged cells. The destroyed and thus necroptotic cells further enhance innate and adaptive immune responses and speed-up the tissue repair process.^3^ After removal of the necroptotic cells by innate (and adaptive) immune cells, the inflammation stops and the immune tolerance toward self-antigens is restored. In addition, the tissue is repaired by a system of replenishment with new cells under the control of tissue-resident stem cells. These stem cells divide asymmetrically upon cell damage or hormonal signals, giving rise to new stem cells that maintain the capacity to divide and form progenitor cells programmed for terminal differentiation. The progenitor cells have the capacity to divide several times before they differentiate into mature terminal tissue cells that lack the capacity to further proliferate.^4^ Aging of body cells, including those of the immune system, increases the risk of aberrant immune responses that may result in the development of immune-related diseases. This occurs especially in individuals that carry mutations in genes associated with immunity, e.g. in individuals that are genetically predisposed to developing immune-related diseases.^5,6^

## Autoimmune diseases

In the case that a repair response remains active because the immune tolerance is not restored in a timely manner, the process becomes self-sustaining and chronic.^7^ Wound healing is a beneficial process, but chronic self-sustaining wound healing is detrimental to the affected tissue and may result in the development of an autoimmune disease. In this process the primary targets of the chronically or repeatedly stimulated T cells, e.g. the primary self-antigens (pSAgs) are driving the pathogenesis and thus disease progression. Relatively late in the disease process when the affected tissue is chronically inflamed, secondary self-antigens presented in a proinflammatory context also become targeted by activated T cells. The gradual increase in the number of targeted self-antigens is named epitope spreading.^8^ In contrast to pSAgs that are produced exclusively in the tissue/organ affected by the autoimmune disease, secondary self-antigens usually are produced throughout the body. Degenerative/inflammatory diseases resemble autoimmune diseases in which a tissue/organ is damaged by sustained or flaring T cell responses directed to tissue-specific pSAgs. Autoimmune diseases are caused by either rare genetically determined cellular defects (the monogenetic or familial cases) or much more frequently by environmental responses (the sporadic or idiopathic cases) in those genetically predisposed to the disease.^8^ The list of autoimmune diseases is rapidly growing beyond the established ‘classic’ autoimmune diseases such as diabetes mellitus type 1 (DM1), arthritis and multiple sclerosis (MS) to encompass other even more important diseases. These diseases include neurodegenerative diseases such as Alzheimer’s dementia (AD) and Parkinson disease (PD), psychiatric diseases such as schizophrenia, and depression, and certain metabolic diseases such as obesity and diabetes mellitus type 2 (DM2), chronic obstructive pulmonary disease (COPD), inflammatory bowel diseases (IBD), atherosclerotic cardiovascular disease (ACD) and many more.^8–14^

To date, autoimmune diseases have no approved cures and for most of them symptomatic or disease masking treatments are the only therapies available. Disease-specific inhibition of the destructive immune response would be the ultimate solution for this group of diseases as it leaves the patient’s immune system intact. This can be achieved by tolerization, in which the immune response to the pSAg of the disease is reversed into a pSAg-specific immune tolerance response. Restoration of the immune tolerance to the pSAgs of autoimmune diseases will stop the tissue destruction by the antigen specific T cells and halt disease progression. This has been attempted by administration of purified disease-specific pSAgs or derived peptide fragments to patients. However, naked proteins or peptides are rapidly degraded in the body. Linkage of peptides to nanoparticles to improve their stability results in a transient therapeutic effect with a narrow spectrum of activity. It remains questionable whether this therapeutic activity in mice can be repeated in clinical settings.^15,16^

In order to develop effective antigen-specific treatments for autoimmune diseases we need to know the disease specific pSAgs. Our knowledge on the immunology of most of the newly identified autoimmune diseases is fragmented and for most autoimmune diseases only candidate pSAgs are known.^8^ In patients with an autoimmune disease it is almost impossible to identify the pSAgs of the disease, since the characteristic disease symptoms resulting from the tissue damage manifest often years after the onset of the sustained destructive immune responses. Furthermore, it is difficult if not impossible for most of the diseases to obtain samples of the chronically inflamed tissue needed to characterize the immune responses, this is especially the case for neurodegenerative diseases for which, in most cases, accurate diagnosis can only be confirmed post mortem. For these reasons, most efforts to identify the pSAg of an autoimmune disease have been dedicated to characterizing antibody responses in patients with the disease.^17^ These studies have revealed the presence of a plethora of auto-antibodies in the serum of patients that, with a few exceptions, bind to intracellular components including enzymes that are released in the blood after cell destruction. The extracellular enzymatic activity may result in the accumulation of modified and immunogenic protein deposits in the inflamed tissues, providing a new source of antibody antigens.^18^ Because the studies are performed with blood or tissue samples from patients that have had the disease for a long time, most of the identified antibody targets represent secondary self-antigens, which severely challenges our ability to uncover the pSAg of the disease.

The role of humoral responses in the development of autoimmunity has remained unclear. For most if not all autoimmune diseases the pathogenesis is driven by T cell responses to pSAgs. The causal relationship between chronic T cell activation directed to the pSAg of a disease and the disease symptoms has been demonstrated in experimentally induced animal models. Studies using these inducible animal models revealed that the pSAg of an autoimmune disease is predominantly or exclusively expressed in the affected tissue and that all patients have a T cell response to it. In addition, induction of a T cell response to the pSAg in an animal by vaccination results in the development of the disease. To date, the vaccination-based autoimmune disease models rely on subcutaneous or intramuscular administration of animals with purified self-antigen or peptides derived together with a complete Freund’s adjuvant (CFA). This method is expensive, labor-intensive and the life-long presence of CFA in the body of the injected animals complicates the subsequent testing of interventions to the disease in the symptomatic animals. Some autoimmune diseases can be induced in mice by intramuscular administration of an expression plasmid encoding the pSAg of the disease.^19,20^ However, intramuscular pSAg expression from expression plasmids does not result in the development of disease symptoms for most of the autoimmune diseases. To develop effective treatments for autoimmune diseases, more detailed knowledge is needed of the pathogenesis of specific diseases followed by identification of the pSAgs. These objectives can only be met by studying pathogenic processes using animal models of autoimmune diseases. Since most of the autoimmune diseases are sporadic, inducible animal disease models are preferred above wild type or monogenetic animal models.

## Viral vectors to induce immune tolerance

Since viruses evolved to deliver and express their genetic information in host target cells, viral vectors are by far the most effective gene delivery vehicles to express self or foreign proteins in vivo (see Table 1). Hosts on the other hand, evolved effective antiviral mechanisms to prevent and limit infection. Host cells use membrane-bound toll-like receptors (TLRs) and cytoplasmic RIG-I-like receptors (RLRs) to sense PAMPs associated with invading viruses. Intracellular TLRs bind viral genomes and extracellular TLRs bind certain viral structural proteins, whereas RLRs bind virus-specific RNAs that are generated following replication. Activation of TLRs or RLRs leads to the induction of an inflammatory response and ultimately in an adaptive immune response directed to viral antigens.^21^ TLR activation (before replication) usually results in the induction of B cell-mediated antibody responses to viral antigens. For this reason vaccines consisting of non-replicating adjuvated viral structural proteins are able to transiently protect vaccinated individuals from infection by the cognate virus. Vaccination with empty viral capsids or purified viral structural proteins lacking an adjuvant usually are non-immunogenic in vivo. Fully RD viral gene delivery vectors are considered to be harmless components by the immune system and are therefore non-immunogenic when injected in hosts that are naïve for the cognate virus. RLR activation (after replication) is a much stronger proinflammatory signal and results in the induction of predominantly T cell responses to viral antigens. This is the reason why RC attenuated virus strains are extensively used as prophylactic vaccines to provide usually life-long protection to reinfection by the homologous wildtype virus.^22^

**Table 1.** An overview of the viral gene delivery vectors for gene and immunotherapies. RD: replication-defective. RC: replication-competent

Replication Capacity	Cognate virus	Properties	Therapeutic use	Strength	Weakness
RD	Lentivirus (LV)	Stable integration in the chromosomal DNA. Large cloning capacity of ~9 kb.	Genetic disorders Cancer	Stable long-term expression. Transduction of dividing and non-dividing cell types.	*Ex vivo* gene replacement therapies. Potential genotoxicity.
RD	Adeno-associated virus (AAV)	Resides as episome in the nucleus. Cloning capacity of 4.5 kb.	Genetic disorders	No chromosomal integration. Various serotypes available to direct tropism. Suitable for *in vivo* applications.	Immunogenic in humans.
RD	Simian Virus 40 (SV40)	Resides as episome in the nucleus. Cloning capacity of 2.7 kb.	Genetic disorders Autoimmune diseases Allergies	Non-immunogenic in humans. No chromosomal integration. Suitable for *in vivo* applications.	Only one serotype available.
RC	Adenovirus (AdV)	Resides as episome in the nucleus. Large cloning capacity of ~7.5kb.	Infectious diseases Cancer	Various serotypes available to direct tropism. Highly immunogenic.	The immune response is mainly directed against viral proteins, potentially concealing the anti-tumor antigen response. Transient transgene expression.
RC	Herpes Simplex virus (HSV)	Resides as episome in the nucleus. Very large cloning capacity of >30 kb.	Infectious diseases Cancer	Highly immunogenic.	The immune response is mainly directed against viral proteins, potentially concealing the anti-tumor antigen response. Transient transgene expression.
RC	Vaccinia virus (VACV)	Resides as episome in the nucleus. Very large cloning capacity of ~25 kb.	Infectious diseases Cancer	Highly immunogenic.	The immune response is mainly directed against viral proteins, potentially concealing the anti-tumor antigen response. Transient transgene expression.
RC	Alphaviruses	Transient replication in the cytoplasm. Large cloning capacity of >7.5 kb.	Infectious diseases Cancer	Highly immunogenic. Vector genomes do not encode viral structural proteins. Vectors from three different alphavirus species available.	Vector production needs optimization.

Among the RD viral vector systems currently used for gene therapy, lentiviral (LV) vectors derived from the human immunodeficiency virus type 1 and adeno-associated viral (AAV) vectors derived from adeno-associated virus are the most popular. For both vectors it has been shown that they are non-immunogenic or tolerogenic in hosts that are naïve to the cognate virus. LV vectors permanently modify transduced target cells by integrating their viral genomes randomly into the host genome. LV vectors are mainly used for *ex vivo* gene replacement therapy to treat blood-related genetic disorders and cancer (see paragraph: Viral vectors to induce immune responses).^22^ The genomes of AAV vectors remain as stable episomes in the nuclei of transduced target cells and AAV vectors are mainly used for *in vivo* gene therapies. Similar to LV vectors the first AAV-based gene therapies have now reached the market.^23^ The preclinical studies using animal models of genetic disorders revealed that AAV-mediated transgene expression in the muscle is associated with immunity to the transgene protein, whereas that in the liver of animals results in the induction of immune tolerance to the transgene protein.^24,25^ The tolerogenic potential of RD AAV vectors in animals naïve for the cognate virus is crucial for establishing stable and long term transgene expression in the liver.^26^ For the best-studied autoimmune disease animal models (DM1, arthritis and MS) it has been shown that RD viral vector-mediated expression of the pSAg of the disease in the liver both protects and cures the treated animals from the autoimmune disease.^27–29^ The preclinical AAV-based gene replacement and tolerization studies in animals demonstrated that RD viral vector-mediated tolerization has an enormous potential for effectively treating patients with autoimmune diseases.^8,29,30^ However, nobody at that time realized that the animal species used in the AAV studies, such as mouse, rat and dog are immunologically naïve for AAV, which is a primarily human virus that co-replicates with adenoviruses: the causal agents of the common cold. Almost the entire human population has been exposed to AAV and developed a strong immune memory for the viral capsid proteins.^31,32^ Numerous clinical studies using recombinant AAV vectors indeed confirmed that administration of vector particles elicits innate and adaptive immune responses against the viral and transgene-encoded proteins in the majority of treated patients. The immune responses lead to elimination of the transduced cells from the body and decreasing expression levels of the therapeutic transgenes over time, compromising re-administration of the vector.^33^ AAV’s immunogenicity in humans, and as a result its clinical inefficacy, will remain the major challenge for the development and approval of new AAV vector-based interventions. Furthermore, the immunogenicity of AAV vectors in humans is the main reason why tolerization using an RD viral vector has not been tested in the clinic so far. Overall, in order to develop effective *in vivo* gene replacement therapies and to efficiently restore immune tolerance in patients with an autoimmune disease, a new non-immunogenic viral gene delivery vector is needed.

RD simian virus 40 (SV40) vectors are an attractive alternative to AAV vectors for clinical gene therapy. SV40 is a polyomavirus that strictly replicates in its natural host, macaques, where it causes chronic asymptomatic infections. SV40 has a 5.25kb long circular double-stranded DNA genome that encodes two genes. The early gene encodes two non-structural replication-associated proteins Small T antigen (STag) and Large T antigen (LTag). The late gene codes for the structural viral proteins VP1, VP2 and VP3. RD SV40 vectors have thus far been generated by deleting the coding region of the early gene. SV40 particles enter infected cells via the caveolar-endosomal route, but in contrast to other viral vectors are able to avoid lysosomal degradation, thereby evading exposure to the host immune system.^34,35^ Because humans can be considered naive to SV40, it is expected that RD SV40 vectors are non-immunogenic or tolerogenic when applied in humans. The lack of immunogenicity in humans and capacity to induce immune tolerance to transgene proteins render SV40 vectors highly attractive for use in gene replacement and tolerization therapies to treat genetic disorders and autoimmune diseases.^36^

## Cancer

During tissue repair, mutations occur in the genome of dividing (stem and progenitor) cells. Most of these somatic mutations are silent and do not affect cellular functionality. These mutant cells are tolerated by the immune system. However, cells with mutations in oncogenes or tumor suppressor genes that inhibit cell death or cell cycle arrest gain the capacity to divide in an uncontrolled manner.^37^ Such derailed or malignant cells secrete DAMPs that activate innate immunity cells. The activated innate immunity cells destroy the malignant cells, eventually with help from activated T cells that target self-antigens specific for the malignant cells, named primary tumor-associated antigens (TAAs), present on the cellular membranes of the tumor cells.^38^ TAAs are usually encoded by genes expressed early during embryogenesis, in differentiated tissue cells or by genes overexpressed in the tumor.^39^ Malignant cells that are capable of escaping from this stringent immune surveillance further proliferate and undergo genetic and epigenetic changes that result in the expression of a range of secondary tumor-associated neo-antigens (TANAs).^40^ Under the pressure of this immune selection, the malignancies induce strong immune tolerance responses to the TAAs and TANAs and rapidly evolve into immune-tolerated tumors. Under the same selection pressure, genetic and epigenetic changes are responsible for tumors to become resistant to cancer treatments.^41^ Immune-tolerated (and treatment-resistant) tumors have the capacity to expand and metastasize, eventually becoming lethal to the patient.^42^ In patients with autoimmune diseases or chronic virus infections the risk is increased that immune-tolerated tumors emerge in the chronically inflamed tissues/organs compared to that in healthy individuals.^43^ This phenomenon adds to the worldwide growing incidence of immune-related diseases over the last decades.

To date many cancers are effectively treated by surgical removal combined with radio and/or chemotherapy to clear the body of all tumor cells. Chemotherapeutics act relatively non- specifically by targeting rapidly dividing cells and their systemic use is therefore associated with severe side effects. Therapeutic antibodies binding TAAs or immune tolerance promoting (co-inhibitory) molecules on the surface of tumor cells represent a remarkable breakthrough in cancer medicine. However, these immunotherapies are effective in only certain subsets of patients and many patients who initially respond eventually relapse via antigenic escape via antigenic escape.^44–46^

For many rapidly metastasizing cancers, effective treatments have not become available creating a strong unmet medical need. Because of its potential safety, specificity, and long-lasting response, therapeutic cancer vaccination is an attractive method to completely clear the body of all tumor cells. Effective vaccines need to break the immune tolerance to the TAAs or TANAs. Therefore, strong PAMPs and/or DAMPs named adjuvants, co-stimulation and repeated vaccination schedules have been used to amplify the activation and expansion of tumor antigen-reactive T cells. Cancer vaccines consisting of TAA- or TANA-derived peptides supplemented with strong adjuvants or DNA/RNA molecules expressing tumor antigens have shown limited efficacy in clinical studies, just as cellular vaccines consisting of living or inactivated tumor cells, dendritic cells loaded with TAAs or microorganisms expressing TAAs. Apparently, these vaccines are not potent enough to break the immune tolerance to tumor antigens.^47,48^

## Viral vectors to induce immune responses

Many viruses have a preference for infecting rapidly dividing cells. Oncolytic viruses are oncotropic nonpathogenic viral strains that preferentially kill (rapidly dividing) malignant cells. Adeno-, herpes-, reo-, retro-, picorna-, rhabdo-, paramyxo-, parvo-, poxviral strains have been extensively tested for their capacity to selectively destroy tumor cells.^49^ However, several limitations prevent the use of most naturally occurring viruses for oncolytic virotherapy. Many of them are immunogenic and usually rapidly inactivated/removed from the body by the patients’ immune system. Moreover, oncolytic viruses have a limited capacity to induce tumor-directed T cell responses. In order to generate oncolytic viral strains with superior clinical efficacy oncolytic RC viral vectors were generated that encode immune-stimulatory proteins or proteins that lure the immune response to tumors instead of the vector.^50^ Despite such enhancements, the induced T cell responses are generally not strong enough to break the immune tolerance to the tumor-antigens.

LV vectors expressing tumor-specific T cell receptors (TCRs) or chimeric antigen receptors (CARs) that combine the properties of T cell receptors with the specificity of antibodies are used to transduce T cells isolated from the blood of cancer patients. The transduced T cells are re-infused in the blood stream of the autologous patients, where they bind and subsequently destroy cancer cells. To date, CAR T cell therapies specific for B cell surface molecules have reached the market for the treatment of B cell leukemias and lymphomas. Although highly successful, a major disadvantage of using LV vectors for T cell therapies is that the CAR-positive T cells remain permanently in the bloodstream of treated patients, continuously removing body cells that express tumor antigens at their surface.^51^ LV vector-based T cell therapies targeting self-proteins may therefore cause autoimmune diseases. This problem can be solved by using RD viral vectors that do not integrate in the genome but remain in the nuclei of transduced T cells as stable episomes. It is expected that after a number of cell divisions the tumor cells have been removed, whereas the CAR-T-expressing vector molecules will dilute-out and the development of autoimmune disease symptoms will be prevented (see paragraph: Viral vectors to induce immune tolerance).

It has been reported that RC viral vectors encoding tyrosinase TRP-2, one of the melanoma TAA’s is capable of breaking the immune tolerance to the TAA, thereby inducing melanoma destruction in mice. Simultaneously, the induced T cell response to TRP-2, but not those to other melanoma TAA’s, induces the development of vitiligo, an autoimmune skin depigmentation disease.^47,52^ These experiments clearly demonstrate that TRP-2 is a relevant melanoma TAA and the pSAg of vitiligo, implying that anti-tumor immune responses and autoimmune responses are identical processes.^53^ RC viral vectors encoding a tumor antigen effectively break the immune tolerance to the antigen and thereby induce tumor destruction by activated T cells, whereas those encoding a pSAg of an autoimmune disease effectively induce autoimmune disease symptoms. Vaccines based on RC and thus self-adjuvating viral vectors have been shown to be highly effective in inducing cellular immune responses in vaccinated animals.^54^ The best studied tumor antigen-encoding RC viral vectors for use as cancer vaccines are derived from adeno-, herpes- and poxviruses. These viruses have large double-stranded DNA genomes and their RC vector derivatives encoding tumor antigens express many viral proteins in transduced cells. As a consequence, a major part of the induced T cell responses is directed to viral proteins and not to the tumor antigens. Upon introduction in a host the vectors are rapidly inactivated by T cells and repeat vaccination will not enhance the anti-tumor antigen immune response.^55^ To overcome this limitation, prime-boost vaccination schemes have been used where a tumor antigen is delivered with one RC viral vector first, followed by a boost with the same tumor antigen delivered by a different viral vector or other therapeutic (e.g. adjuvated peptide, expression plasmid, antibody). Unfortunately, to date cancer vaccines involving RC adeno-, herpes- and poxviral vectors are not powerful enough to break the immune tolerance to tumor cells.^56–58^

RC vectors derived from RNA viruses, in particular those which lack the coding capacity for the viral structural proteins are attractive alternative cancer vaccine carriers to prevent anti-vector immunity. In this respect Alphaviruses, including Semliki Forest virus (SFV), Sindbis virus (SINV) and Venezuelan equine encephalitis virus (VEEV), meet these prerequisites. Alphaviruses have single stranded RNA genomes harboring two coding domains or cistrons. The first cistron is translated from the genomic RNA and encodes the non-structural proteins involved in viral RNA replication and transcription. The second downstream cistron is translated from a sub-genomic RNA that is transcribed from a sub-genomic promoter by the viral non-structural proteins and encodes the structural proteins. Expression plasmids have been generated that encode RC Alphaviral RNAs in which the second cistron is replaced by a gene-of-interest. Intradermal or intramuscular administration in animals with plasmid-based RC Alphaviral vectors results in the induction of strong T cell responses to the transgene proteins, while anti-vector immune responses are limited. Vaccination studies with plasmid-based Alphaviral vectors encoding TAAs have successfully demonstrated tumor regression and protection against challenges with tumor cells in animal tumor models.^59^ SFV- or VEEV-based Alphaviral vector particles encoding TAAs including the human papillomavirus E6,7 non-structural protein, the prostate specific membrane antigen PSMA or HER2/neu have been tested in clinical studies. These studies revealed that RC Alphaviral vector vaccines induce cellular cytotoxicity against tumor cells coinciding with longer overall survival of the treated patients.^60–62^ To further enhance the therapeutic potential of RC Alphaviral vector vaccines, prime boost vaccination schemes combining different Alphaviral vectors (SFV, SINV or VEEV) encoding the same tumor antigen could be applied.

Besides being highly promising cancer vaccine carriers, RC Alphaviral vectors are well suited to identify pSAgs of autoimmune diseases and to develop reliable inducible animal models for this group of diseases. The coding sequences of these proteins can be inserted in RC Alphaviral vector plasmids an can be directly administered with the recombinant plasmid DNA into mice. This fast and convenient method to induce autoimmune disease symptoms in mice will lay the foundation to increase our knowledge of the pathogenesis of autoimmune diseases and will serve as the starting point for developing effective therapeutic interventions.

The major diseases of our time, including cancer, autoimmune diseases and allergies are caused by aberrant immune responses to self or non-self components. With our rapidly increasing knowledge of gene therapy, vaccinology, virology and immunology we now have RC and RD viral vector tools available to effectively address immune-related diseases. These vector systems are perfectly suited to unraveling the pathogenesis of immune-related diseases and developing the next generation of prophylactics and therapeutics to combat this cluster of diseases with a high impact on our society.

In the next few years further research using RC Alphaviral vectors that effectively break the immune tolerance to self-antigens of autoimmune diseases will result in the development of inducible animal models for human autoimmune diseases. Such animal models will be highly useful for studying the disease pathogenesis and testing novel prophylactics and therapeutics. Moreover, RC Alphaviral vectors expressing TAAs or TANAs have the potential to serve as effective cancer vaccines. Within five years from now it is expected that new Alphaviral vector-based cancer vaccination approaches will be tested for safety and efficacy in clinical settings. Because of their lack of immunogenicity and capacity to induce immune tolerance to transgene proteins in humans, RD SV40 vectors are well positioned to become the viral vector system of choice for developing effective gene replacement and immunotherapies to treat genetic disorders, autoimmune diseases and allergies. To date, SV40 vectors have not been tested in clinical studies. Considering the potential of this vector system a clinical study of an SV40-based therapeutic is highly desired to demonstrate the safety and efficacy of this vector system in humans.

## Conclusions

It is predicted that with our aging population, health care costs will rise dramatically in the decades to come.^63^ In order to keep the health care expenditure sustainable, we desperately need effective and preferably curative treatments for the main chronic age- and immune-related diseases including cancer and autoimmune diseases. Recent developments in gene therapy and vaccinology have opened the way to develop effective treatments for this seemingly incurable group of diseases. Powerful viral gene delivery vectors have recently become available that have the potential to generate the platforms of a whole new generation of medicines based on their high efficacy to induce or suppress immune responses against self and foreign proteins. RC Alphaviral vectors can be used to develop effective immunotherapies for cancer, whereas RD SV40 vectors can be employed in tolerization therapies for autoimmune diseases including neurodegenerative & psychiatric diseases, ACD, obesity, diabetes mellitus, arthritis, COPD and many other inflammatory diseases. As an important first step in the development of effective tolerization strategies the pSAgs of individual autoimmune indications need to be identified. Together, Alphaviral and SV40 vector platforms have the potential to develop effective interventions for immune-related disease. Interventions that will contribute significantly to the healthy aging concept and dramatically improve the life expectancy and quality of life of individual patients.
